# Safety Learning in Anxiety, Pavlovian Conditioned Inhibition and COVID Concerns

**DOI:** 10.3389/fpsyg.2022.866771

**Published:** 2022-04-04

**Authors:** Meghan D. Thurston, Helen J. Cassaday

**Affiliations:** ^1^School of Psychology, University of Nottingham, Nottingham, United Kingdom; ^2^Department of Clinical Psychology, The Royal Derby Hospital, Derby, United Kingdom

**Keywords:** anxiety, conditioned inhibition, COVID-19 pandemic, safety learning, cognitive behavioral therapy

## Abstract

Experimental studies of fear conditioning have identified the effectiveness of safety signals in inhibiting fear and maintaining fear-motivated behaviors. In fear conditioning procedures, the presence of safety signals means that the otherwise expected feared outcome will not now occur. Differences in the inhibitory learning processes needed to learn safety are being identified in various psychological and psychiatric conditions. However, despite early theoretical interest, the role of conditioned inhibitors as safety signals in anxiety has been under-investigated to date, in part because of the stringent test procedures required to confirm the demonstration of conditioned inhibition as such. Nonetheless, the theoretical implications of an inhibitory learning perspective continue to influence clinical practice. Moreover, our understanding of safety signals is of additional importance in the context of the increased health anxiety and safety behaviors generated by the COVID-19 pandemic.

## Introduction

Fear and anxiety are normal, albeit subjectively negative emotions and motivations, that arise in response to perceived threat, and which are typically experienced under particular circumstances. Fear has been distinguished from anxiety as more phasic (time-limited) state, whereas the term anxiety is used to refer to a tonic state of readiness (Adolphs, 2013). Like fear, anxiety can be restricted to specific situations in state anxiety, but high trait anxiety reflects a more general tendency or predisposition to perceive a wider range of situations as threatening. In either case, adrenalin is released to help the individual prepare with the fight-flight response, which is reflexive and hardwired in phylogenetically old areas of the midbrain, and includes for example increased heart rate in readiness for action.

This perspective article first outlines the theoretical framework provided by evolutionary perspectives on anxiety. We then focus on recent evidence confirming the importance of safety learning for cognitive behavioral approaches in cases of anxiety. Third, we consider the theoretical context provided by animal learning studies. In conclusion, we consider the implications of our understanding of safety learning for the mental health impact of the developing (and sometimes rapidly changing) situation presented by the COVID-19 pandemic.

### Evolutionary Perspectives on Anxiety

In general, we can be slow to learn to fear new objective threats such as those arising on our roads or the health effects of cigarette smoking and excess alcohol consumption. More common causes of fear and anxiety make better sense from a phylogenetic rather than a learning perspective. However, the rapid spread of COVID-19 infections should have triggered our phylogenetic predispositions. Fear of infection is evolutionarily prepotent and prepared, hence also automatic and resistant to cognitive control (Öhman and Mineka, 2001). Moreover, adaptive avoidance of illness and death produced by infectious disease, mediated by feelings of disgust as well as fear, can be seen to provide us with a proactive behavioral immune system, complementary to the reactive defences provided by the physiological immune system (Troisi, 2020).

Within an evolutionary framework, the fight-flight response has been suggested to be a key component of a survival optimization system, involving a cascade of cognitive and affective responses to threats of increasing magnitude (Mobbs et al., 2015). Without doubt, fear can be adaptive to the extent the resulting anticipatory responses are proportionate to the level of environmental threat. However, we need to understand the functional role of fear and anxiety in relation to the environmental context (Adolphs, 2013). Actions that lead to the removal of valid threats enhance our survival and are reinforced, as well as selected for evolutionarily. Indeed taking the evolutionary perspective has already underscored the point that too little anxiety accompanied by the loss of normal defensive responses would also be harmful, amounting to *hypo*phobic disorder (Marks and Nesse, 1994).

Conversely, it is well-recognized that excessive anxiety impacts wellbeing. For example, adrenalin causes a range of physiological changes which typically include sweating, heavy breathing and shaking as well as increased heart rate. Moreover, for some individuals these physical changes are over-interpreted, resulting in further increased anxiety, which is experienced as aversive and thus leads to the development of avoidance behaviors. To the extent these behaviors are disproportionate to the objective level of threat, and the underlying fear is irrational and excessive, there is the risk of anxiety disorders and maladaptive avoidance behaviors, undermining our health and well-being. From a clinical perspective, how should we in practice judge when levels of anxiety and the resulting anticipatory avoidance responses are proportionate to the level of environmental threat and hence adaptive? For example, in the circumstances of the COVID-19 pandemic, where the threat of serious illness is real, the failure to adopt appropriate safety behaviors can be maladaptive, potentially endangering health and even survival.

The theoretical context to the study of fear conditioning and safety learning is key to the nature of the inhibitory processes in play and now highly topical in the context of the COVID-19 pandemic. Fear of infection with COVID-19 has understandably greatly increased health anxiety and safety behaviors, in some cases perhaps disproportionately. On the other hand, individuals failing to adopt appropriate safety behaviors may have put themselves and their close contacts at risk. Pre-pandemic approaches to managing fear, anxiety and avoidance behaviors, presupposed that the wider environment was objectively safe. Now our presumptions to the effect that such anxiety-related behaviors should be eliminated using cognitive-behavioral therapy, must be constantly re-evaluated in relation to the objective health risks posed by COVID-19, in different phases of the pandemic, and in relation to the financial and social costs of COVID-19 safety behaviors for other aspects of our well-being.

### Safety Learning in Anxiety

Although there are effective treatments, the underlying mechanisms of the various anxiety disorders are still not fully understood and aetiological models are multi-factorial. Such models incorporate specific environmental triggers, ranging from discrete cues such as dirt, to the diffuse range of stimuli engendered by social activities (Ehlers and Clark, 2000; Rachman, 2012). Here, we focus on trigger cues and the counter-cues provided through safety learning.

Controlled animal studies confirm that safety signals moderate stress reactivity, buffering the effects of uncontrollable stressors (Christianson et al., 2012). Patients with anxiety disorders report awareness of a variety of safety signals, from the presence of a trusted individual, to the availability of their phone and medications (Craske et al., 2014). Health-related specific phobias are also characterized by safety behaviors such as apparently excessive medical check-ups, body scanning, excessive cleaning or other hygiene-related safety behaviors such as the use of sanitized wipes (Rachman, 2012). Yet paradoxically *deficient* safety discrimination has been reported to characterise individuals high in trait anxiety (Gadenzam et al., 2013; Haaker et al., 2015) and some patients with anxiety disorders (Duits et al., 2021).

The evidence-based recommended choice of treatment for anxiety disorders is cognitive behavioral therapy. This approach focuses on breaking identified associations between maladaptive cognitions and unwanted behaviors. Notably patients may be specifically encouraged to drop safety behaviors. For example, a driver with who after an accident excessively checked his mirrors would be encouraged to return to his pre-accident driving pattern (Wells et al., 2016). Clinically there is some mixed evidence as to the role of safety signals in the progress of exposure treatments, but the gradual removal of safety signals is the generally favored approach. Removal may be combined with strategies to promote the recognition and consolidation of the non-occurrence of the expected aversive event, for example using discussions with the therapist to encourage reflection on the absence of the feared outcome (Craske et al., 2014).

### Pavlovian Conditioned Inhibition

**Figure 1:**
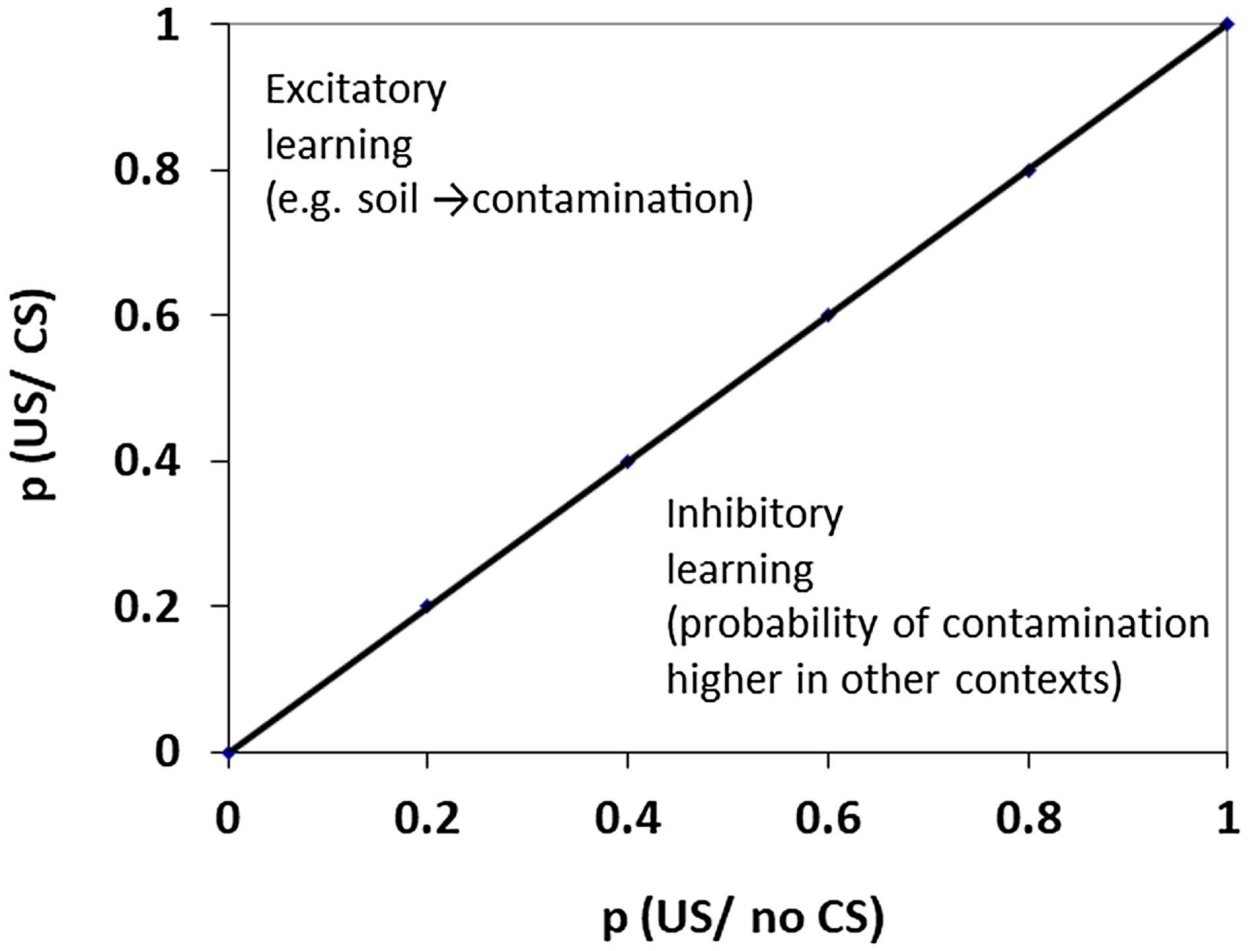
Positive prediction error is seen when the probability (*p*) of the unconditioned stimulus (US) is increased on presentation of the CS+ and generates excitatory conditioning. Negative prediction error is seen when the probability of the US is decreased on presentation of the CS- and generates inhibitory conditioning. When *p* (US/CS) = *p* (US/no CS) along the diagonal trend line there can be no new learning. In the example shown, soil provides excitatory stimuli that generate fear of contamination. However, the stimuli provided by exposure to soil could become inhibitory (safety) signals if the probability of contamination were perceived as higher in contexts where soil-related cues are absent (in which case gardening might feel like a safer exercise than swimming at the public baths). Positive and negative prediction error are depicted as Pavlovian phenomena arising from specified and predictable relationships between (CS and US) environmental events. In addition, stimuli generated by instrumental responses (both external and internal to the agent) can have Pavlovian properties, for example successful avoidance responses generate inhibitory (safety) signals.

The establishment of safety cues provides an example of Pavlovian inhibitory learning (Figure 1). In order to demonstrate that conditioned inhibition has occurred and that the cue presumed inhibitory is a true inhibitor, responding to this unreinforced CS- must be clearly distinguishable from that elicited by the equivalent stimulus in the reinforced CS+ role. Summation and retardation tests have been considered definitive. The summation test involves taking a CS+, pre-trained but not previously presented in conjunction with any CS-, and examining the reduction in excitatory responding seen when this CS+ is now paired together with an established CS- (pre-trained in conjunction with a different CS+). A true inhibitor should reduce the responding which would otherwise be elicited by any subsequent CS+ for the same unconditioned stimulus (US) with which it is subsequently paired. The retardation test similarly relies on the notion that a CS- develops negative associative strength. Retardation is assessed when the previously trained inhibitor (CS-) is now paired with a US. Compared with the rate of acquisition seen with a neutral CS, conversion to a CS+ is slowed or retarded for a CS-.

The two test method to confirm conditioned inhibition (by both summation and retardation tests) has been widely adopted in animal studies, but has been harder to implement in human studies. In human studies, there have been some reports of conditioned inhibition confirmed by summation test (He et al., 2013; Heym et al., 2014; Thurston and Cassaday, 2015) but these have been relatively few (Sosa and dos Santos, 2019; Sosa and Ramίrez, 2019). Indeed, it has been argued that studies of feature negative inhibitory discrimination learning of the kind typically used to successfully demonstrate conditioned inhibition, but which do not include (or do not subsequently pass) the formal tests to confirm conditioned inhibition should also be considered (Sosa and dos Santos, 2019; Sosa and Ramίrez, 2019). Feature negative discriminations of the form A+/AX- introduce the conflict engendered by responding or refraining to respond, depending on the presence or absence of the inhibitory cue (X), and on this basis can be considered a good proxy for conditioned inhibition.

Translational studies of inhibitory learning have provided new insights into improving the efficacy of exposure-based interventions, particularly those which explicitly include response prevention. Laboratory studies of fear extinction suggest that the associations are not eliminated during such treatments but rather exposure is effective because of the learning of new competing associations. Where non-threat is signalled these associations are inhibitory (Jacoby and Abramowitz, 2016). Inhibitory learning approaches to exposure therapy assume that the originally aversive CS enters into competing associations, now with the absence of the feared unconditioned stimulus (US) through extinction learning (Bouton et al., 2001; Craske et al., 2014). This line of thinking generates the perhaps paradoxical prediction that exposure therapy should be more effective when multiple fear-provoking cues are combined to increase the discrepancy when the predicted outcome does not in fact occur (Craske et al., 2014; Jacoby and Abramowitz, 2016).

### Instrumental Avoidance Learning

Beyond their role in the narratives of the mental landscape, from awareness of contingencies and the accompanying feelings, simple associations drive actions. The focus of this perspective has been on Pavlovian (respondent) rather than instrumental (operant) conditioning, and Pavlovian studies of conditioned inhibition have not been designed with an instrumental avoidance response in mind. However, avoidance learning has been extensively studied in animal experiments, usually conducted using laboratory rats, which when given a signal of foot shock rapidly learn to make a response to avoid experiencing any further shock. The two-process theory of avoidance proposed that anticipatory anxiety is initially learned through Pavlovian conditioning of the signal for foot shock. Through the second process, instrumental learning, negatively reinforced avoidance responses are produced in response to the anxiety signal (Mowrer, 1956). This theory was later developed to include a key role for the safety signals generated by the successful execution of an avoidance response, through consideration of the likely role of inhibitory learning (Gray, 1975). There is experimental evidence to show how a safety signal may protect the classically conditioned fear response from extinction and thus account for the persistence of the avoidance behavior (Soltysik et al., 1983). Moreover, safety signals have been shown to be emotionally “relieving” in such studies: rats showed a strong correlation between the rate of deep breaths (“sighs of relief”) and the presentation of the signal for the non-occurrence of the otherwise expected shock (Soltysik and Jelen, 2005). Similarly, in a free-operant lever press avoidance procedure, rats preferentially responded on the lever that produced an explicit safety signal (in addition to the omission of the footshock) as feedback (Fernando et al., 2014). Thus, in addition to the basic behavioral responses, effective avoidance learning can include further actions and thoughts to ease anxiety such that the individual can remain in and cope with the situation. In other words, successful avoidance generates safety signals which inhibit anxiety.

**Figure 2:**
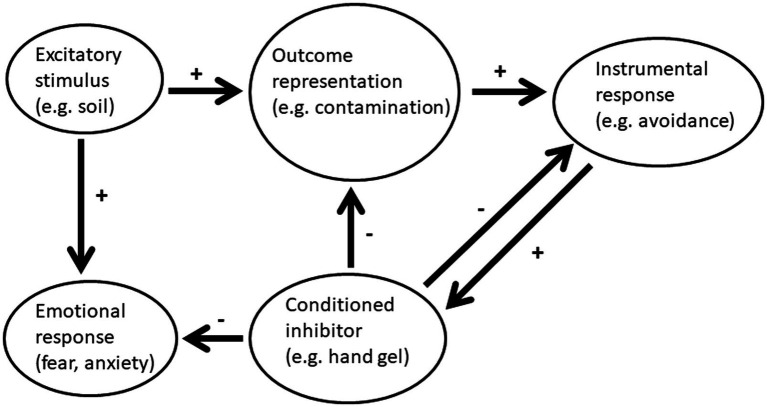
After Pavlovian conditioning the excitatory stimulus elicits not only a conditioned emotional response but also an outcome representation. In turn, through Pavlovian-instrumental transfer, the rate of an ongoing instrumental response can be moderated. If conditioned inhibitors are assumed to act by depressing the outcome representation this can explain both their effects in summation and retardation tests as well as their capacity to reduce Pavlovian-instrumental transfer. In the example shown, the excitatory stimulus provided by exposure to soil results in an outcome representation of contamination which in turn increases avoidance of outdoor activities; however, antibacterial hand gel cues (e.g., the feel of the container in the pocket) can effectively inhibit the outcome representation, reducing both the fear of contamination and the associated avoidance responses. Thus, in addition to the capacity to generate inhibitory (safety) signals, instrumental avoidance responses are bi-directionally controlled by Pavlovian excitors and inhibitors.

Simple associations drive actions and both Pavlovian (respondent) and instrumental (operant) learning mechanisms should be considered (Rajaraman et al., 2022). The link between fear-related associations and actions has been further explored in studies of Pavlovian to instrumental transfer. This term refers to the experimental procedure and underling phenomenon whereby a Pavlovian CS influences instrumental actions by increasing or decreasing motivational salience (Cartoni et al., 2013). In the example shown in Figure 2, the stimuli provided by exposure to stimuli such as those produced by exposure to soil result in an outcome representation of contamination which may in turn result in the avoidance of activities likely to increase the risk of contamination; however, the availability of an antibacterial hand gel in the pocket can effectively inhibit the outcome representation, reduce the fear of contamination and suppress the associated avoidance responses.

Avoidance behaviors are known to be very persistent and are negatively reinforced through the decrease in anxiety and relief from perceived threat. Neutral stimuli that accompany successful avoidance responses are in effect trained as conditioned inhibitors. Similarly, feedback from the successful execution of an avoidance response generates stimuli which reliably predict US omission. Thus theoretically, avoidance can also be secondarily reinforced by the safety signals and hence fear reduction generated by the instrumental response (Cook et al., 1987; Dinsmoor, 2001). Thus, when avoidance responses are possible, more complex Pavlovian-instrumental interactions also emerge. In addition to the capacity to generate inhibitory (safety) signals, instrumental avoidance responses are bi-directionally controlled by Pavlovian excitors and inhibitors (Figure 2).

When the need to generate safety signals, through safety behaviors, starts to impinge on normal daily routines this can suggest the presence of an anxiety disorder: the anxiety disorders are as much a group of behavioral disorders characterized by unwanted actions as cognitive disorders characterized by unwanted thoughts. Indeed, obsessive–compulsive disorder (OCD) has been suggested to arise from excessive stimulus–response habit formation resulting in enhanced avoidance habits (Gillan et al., 2014). Impulsivity in the wider sense also features inhibition deficits and conditioned inhibition has started to be used to characterize components of these deficits (Sosa and dos Santos, 2019).

### COVID Concerns

To our knowledge, the safety learning approach has yet to be applied to our understanding of the mental health impact of the COVID-19 pandemic. However, its relevance is self-evident. People may initially wash and sanitize their hands to reduce the risk of COVID-19 infection, but the safety signals provided by the smell of soap and alcohol will come to secondarily reinforce these behaviors. Similar arguments apply to mask wearing and social distancing, and it may be difficult to reverse these now ingrained behaviors as the risk reduces. As might be expected, individuals with OCD have been identified as particularly susceptible to the mental health impact of the COVID-19 pandemic (Jassi et al., 2020), particularly those with contamination-related OCD (Jelinek et al., 2021). Moreover, there is evidence that COVID-19 has resulted in anxiety symptoms and some experimental evidence of attentional bias for virus-related stimuli, related to pre-existing levels of health anxiety in a normal population (Cannito et al., 2020). Trait health anxiety and cyberchondria are risk factors for poor mental health in consequence of the pandemic (Jungmann and Witthöft, 2020). Internet research (e.g., of infection symptoms) and media exposure can amount to safety-seeking behaviors but also generate potential triggers for further safety-seeking behavior depending on how the findings are interpreted (Starcevic and Berle, 2013; Brown et al., 2019; Garfin et al., 2020).

## Discussion

Remote cognitive-behavioral therapy for severe anxiety in response to the pandemic has been found to be variably effective based on the perceived quality of the early interactions and in particular the focus on solutions to problems arising (Malins et al., 2020). We suggest that, granted relaxed restrictions and/or recommendations and relatively low case numbers, cognitive-behavioral interventions for persistent anxiety should include the identification of safety signals and reflection on the broader range of solutions needed to ensure wellbeing in the wider sense. As we have seen, safety signals protect conditioned fear from extinction and their systematic removal has been a focus of therapeutic intervention (Craske et al., 2014).

This situation is of course particularly complex in the case of COVID-19: given the uncertainties surrounding the efficacy of the available vaccines against new variants (which will likely continue to emerge), to continue with an appropriate range and level of safety behaviors may be entirely rational. The costs and benefits of behavioral exposure interventions for individuals experiencing COVID-related anxiety will need continual review in the light of our current understanding of the risks associated with COVID-19 infection, also taking into account individual vulnerability factors unrelated to mental health (Jassi et al., 2020). Individualized precision interventions should also be tailored to the prognostic markers provided by individual inhibitory learning profiles (Duits et al., 2021). Perhaps most pressing is the need to identify whether safety learning is increased or decreased in connection with different kinds of anxiety-related disorders and concerns (Orr et al., 2000; Jovanovic et al., 2010; Duits et al., 2021).

## Data Availability Statement

The original contributions presented in the study are included in the article/supplementary material, further inquiries can be directed to the corresponding authors.

## Author Contributions

All authors listed have made a substantial, direct, and intellectual contribution to the work and approved it for publication.

## Funding

This work was supported by the Biotechnology and Biological Sciences Research Council [grant number BB/S000119/1] and a School of Psychology PhD studentship.

## Conflict of Interest

The authors declare that the research was conducted in the absence of any commercial or financial relationships that could be construed as a potential conflict of interest.

## Publisher’s Note

All claims expressed in this article are solely those of the authors and do not necessarily represent those of their affiliated organizations, or those of the publisher, the editors and the reviewers. Any product that may be evaluated in this article, or claim that may be made by its manufacturer, is not guaranteed or endorsed by the publisher.
